# Stool Microbiome and Metabolome Differences between Colorectal Cancer Patients and Healthy Adults

**DOI:** 10.1371/journal.pone.0070803

**Published:** 2013-08-06

**Authors:** Tiffany L. Weir, Daniel K. Manter, Amy M. Sheflin, Brittany A. Barnett, Adam L. Heuberger, Elizabeth P. Ryan

**Affiliations:** 1 Department of Food Science and Human Nutrition, Colorado State University, Fort Collins, Colorado, United States of America; 2 United States Department of Agriculture-Agricultural Research Service, Soil-Plant-Nutrient Research Division, Fort Collins, Colorado, United States of America; 3 Proteomics and Metabolomics Facility, Colorado State University, Fort Collins, Colorado, United States of America; 4 Department of Environmental and Radiological Health Sciences, Colorado State University, Fort Collins, Colorado, United States of America; University of Illinois, United States of America

## Abstract

In this study we used stool profiling to identify intestinal bacteria and metabolites that are differentially represented in humans with colorectal cancer (CRC) compared to healthy controls to identify how microbial functions may influence CRC development. Stool samples were collected from healthy adults (n = 10) and colorectal cancer patients (n = 11) prior to colon resection surgery at the University of Colorado Health-Poudre Valley Hospital in Fort Collins, CO. The V4 region of the 16s rRNA gene was pyrosequenced and both short chain fatty acids and global stool metabolites were extracted and analyzed utilizing Gas Chromatography-Mass Spectrometry (GC-MS). There were no significant differences in the overall microbial community structure associated with the disease state, but several bacterial genera, particularly butyrate-producing species, were under-represented in the CRC samples, while a mucin-degrading species, *Akkermansia muciniphila*, was about 4-fold higher in CRC (p<0.01). Proportionately higher amounts of butyrate were seen in stool of healthy individuals while relative concentrations of acetate were higher in stools of CRC patients. GC-MS profiling revealed higher concentrations of amino acids in stool samples from CRC patients and higher poly and monounsaturated fatty acids and ursodeoxycholic acid, a conjugated bile acid in stool samples from healthy adults (p<0.01). Correlative analysis between the combined datasets revealed some potential relationships between stool metabolites and certain bacterial species. These associations could provide insight into microbial functions occurring in a cancer environment and will help direct future mechanistic studies. Using integrated “omics” approaches may prove a useful tool in identifying functional groups of gastrointestinal bacteria and their associated metabolites as novel therapeutic and chemopreventive targets.

## Introduction

A healthy gastrointestinal system relies on a balanced commensal biota to regulate processes such as dietary energy harvest [1], metabolism of microbial and host derived chemicals [2], and immune modulation [3]. Accumulating evidence suggests that the presence of microbial pathogens or an imbalance in the native bacterial community contributes to the development of certain gastrointestinal cancers. A causal relationship between gastric cancer and *Helicobacter pylori* has been established [4], leading to the hypothesis that other host-associated organisms are involved in cancer etiology.

An association between colorectal cancer (CRC) and commensal bacteria has been suspected for decades. For example, *Streptococcus infantarius* (formerly *S. bovis*) became diagnostically important after it was recognized that bacteremia due to this organism was often associated with colorectal neoplastic disease [5], [6]. However, early studies associating genera of bacteria with colon cancer risk were limited to culture-based methods that did not reflect the complexity of the gastrointestinal microbiota [7]–[9]. Development of high-throughput sequencing has facilitated detailed surveys of the gut microbiota, and a more thorough and complex colorectal cancer (CRC)-associated microbiome is emerging. Sobhani et al. [10] found that the Bacteroides/*Prevotella* group was over-represented in both stool and mucosa samples from individuals with colon cancer compared to their cancer-free counterparts. They also found that *Bifidobacterium longum*, *Clostridium clostridioforme*, and *Ruminococcus bromii* were underrepresented in samples from these individuals and concluded that a lack of correlation between tumor stage/size with the over-represented populations suggested a contributory role of the bacteria in tumor development. Two additional studies, published concurrently, examined the microbiota present in the tumor mucosa and adjacent healthy tissue of individuals with colon cancer and both studies revealed an overrepresentation of *Fusobacterium spp* [11], [12], while others have revealed an abundance of *Coriobacteria* and other probiotic species [13], [14].

The question remains whether over-representation of particular microbial species in stool and mucosal samples is indicative of a contributory role in the development of CRC or a consequence of the tumor environment. Although a causal role of intestinal biota in CRC development has not been demonstrated, there is evidence to suggest that induction of pro-inflammatory responses by commensals contribute to tumor initiation and development [10], [14]. Production of genotoxins and DNA damaging superoxide radicals are also mechanisms by which commensals can contribute to CRC development [15]. Alternatively, it has been hypothesized that certain probiotic bacteria act as tumor foragers, taking advantage of an ecological niche created by the physiological and metabolic changes in the tumor microenvironment [14].

To clarify the role of intestinal biota in the development of CRC, it will be necessary to move beyond taxonomic over-representation and examine changes in the CRC associated microbiome in a more functional context. One important functional parameter is how commensal organisms contribute to the flux of metabolites and the breakdown of dietary components. Thus, metabonomics, the study of global changes in metabolites in response to biological stimuli [16], is being applied to identify and characterize the functional microbiome that drives metabolic changes associated with different diets, genotypes, and disease states [17]–[19]. Stool metabolite profiles have been validated as a means of assessing gut microbial activity [20] and the current study contributes to the growing list of gut microbes in the CRC microbiome, but also utilizes a metabonomics approach to identify potential microbiome-metabolome interactions.

## Materials and Methods

### Ethics Statement

All individuals provided written informed consent prior to participating in the study. All study protocols were approved by Colorado State University (Protocol numbers 10-1670H and 9-1520H) and Poudre Valley Hospital-University of Colorado Health System’s Institutional Review Boards (Protocol numbers 10-1038 and 10-1006).

### Sample Collection and DNA Extraction

Stool samples were collected from healthy individuals (n = 11) and recently diagnosed colon cancer patients (n = 10) prior to surgery for colonic resection (Table 1-note: not all samples were subjected to all analyses. See Table 1 footnote). Exclusion criteria for all participants included use of antibiotics within two months of study participation, and regular use of NSAIDS, statins, or probiotics. Individuals that reported chronic bowel disorders or food allergies/dietary restrictions were also excluded from the study. Additional exclusion for CRC patients included chemotherapy or radiation treatments prior to surgery. Stool samples were provided for analyses prior to administration of any pre-operative antibiotics or bowel preparation. Samples were transported to the laboratory within 24 hours after collection by study participants. Stool samples were homogenized, and three subsamples were collected with sterile cotton swabs. DNA was extracted from all samples using MoBio Powersoil DNA extraction kits (MoBio, Carlsbad, CA) according to the manufacturer’s instructions and stored at −20°C prior to amplification steps.

**Table 1:** Study participant characteristics.

Participant ID	Health Status	Sex	Age	BMI	Tumor Stage	Size (cm)	Tumor Location
**CRC1** ^s^	Cancer	M	84	43.3	T2	5	Rectum
**CRC2** ^s,t,g^	Cancer	M	67	39.1	T2	1	Ascending
**CRC3** ^s,t,g^	Cancer	M	47	36.2	T1	0.01	Sigmoid
**CRC4** ^s,t,g^	Cancer	M	68	28.8	T2	2.8	Rectum
**CRC5** ^s,t,g^	Cancer	M	85	26.2	T3	5.5	Ascending
**CRC6** ^t,g^	Cancer	F	51	34	T1	0.5	Sigmoid
**CRC7** ^s,t,g^	Cancer	M	74	28.6	T3	4.5	Sigmoid
**CRC8** ^t,g^	Cancer	F	55	21.6	Tis	4.5	Sigmoid
**CRC9** ^s,t,g^	Cancer	M	30	24.3	T3	1.7	Rectum
**CRC10** ^s,t,g^	Cancer	M	76	26.2	T3	2.5	Ascending
**H1** ^s,t^	Healthy	M	39	24.7			
**H2** ^s,t,g^	Healthy	F	36	22.8			
**H3** ^t,g^	Healthy	F	54	23.8			
**H4** ^t,g^	Healthy	F	57	23			
**H5** ^s,t,g^	Healthy	F	26	35.7			
**H6** ^s,t,g^	Healthy	F	24	24.9			
**H7** ^t,g^	Healthy	F	34	25.2			
**H8** ^s,t,g^	Healthy	M	67	30.1			
**H9** ^s,t,g^	Healthy	M	34	21.9			
**H10** ^s,t,g^	Healthy	F	25	20			
**H11** ^t,g^	Healthy	F	52	26.4			

Sample included in ^s^454 pyrosequencing analysis; ^t^ targeted analysis of bacterial SCFA’s, and ^g^global metabolite profiling by GC-MS. Tis: Carcinoma in situ: intraepithelial or invasion of lamina propria; T1: Tumor invades submucosa; T2: Tumor invades muscularis propria; T3:Tumor invades through muscularis propria into the subserosa or into nonperitonealized pericolic or perirectal tissue.

### Pyrosequencing Analysis

Amplification of the V4 region of the bacterial 16S rRNA gene was performed in triplicate using primers 515F and 806R labeled with 12-bp error correcting Golay barcodes [21]. Twenty µl reactions containing 5 Prime Hot Master Mix (5 Prime, Inc., Gaithersburg, MD) were amplified at 94°C for 5 minutes followed by 35 cycles of 94°C for 1 min, 63°C for 1 min, and 72°C for 1 min followed by a final extension at 72°C for 10 minutes. Replicate PCR reactions from each sample were combined and gel purified using the GenElute Gel Extraction kit (Sigma-Aldrich, St. Louis, MO), followed by an additional purification with AMpure beads (Beckman Coulter, Indianapolis, IN) and quantified with the PicoGreen DNA Assay (Invitrogen, Carlsbad, CA, USA) prior to library pooling. Pyrosequencing was performed under contract by the University of South Carolina’s Engencore Sequencing Facility using a 454 Life Sciences GS FLX System with standard chemistry.

All sequence read editing and processing was performed with Mothur Ver. 1.25 [22] using the default settings unless otherwise noted. Briefly, sequence reads were (i) trimmed (bdiff = 0, pdiff = 0, qaverage = 25, minlength = 100, maxambig = 0, maxhomop = 10); (ii) aligned to the bacterial-subset SILVA alignment available at the Mothur website (http://www.mothur.org); (iii) filtered to remove vertical gaps; (iv) screened for potential chimeras using the uchime method; (v) classified using the Green Genes database (http://www.mothur.org) and the naïve Baysian classifier [23] embedded in Mothur. All sequences identified as chloroplast were removed; (vi) sequences were screened (optimize = minlength-end, criteria = 95) and filtered (vertical = T, trump = .) so that all sequences covered the same genetic space; and (vii) all sequences were pre-clustered (diff = 2) to remove potential pyrosequencing noise and clustered (calc = onegap, coutends = F, method = nearest) into OTUs [24]. To remove the effect of sample size on community composition metrics, sub-samples of 1250 reads were randomly selected from each stool sample. After clustering sequence reads into OTUs (i.e., nearest-neighbors at 3% genetic distance) or phylotypes (i.e., sequences matching a common genus in the Green Genes Database), the replicate sub-samples were averaged to yield a single community profile for each sample. Sample size independent values for alpha diversity community descriptors such as observed species richness (*S_obs_*), Chao1 estimates of total species richness (*S_Chao_*), Shannon’s diversity (*H’*) and evenness (*E_H_*), and Simpson’s diversity (1-D) and evenness (E_D_) were determined by fitting a 3-parameter exponential curve [*y* = *y0*+ *a*(1-e*^−bx^*)] to rarified parameters over a range of 100 to 1250 sequence reads, where the asymptotic maxima is equal to the sum of *y0* and *a*. Effective number of species were calculated as S_H_ = exp (H’) for the Shannon’s index and S_D_ = 1/D for Simpson’s. All sequence data is publicly available through the Sequence Read Archive (SRA) under study accession number ERP002217, which is available at the following link: http://www.ebi.ac.uk/ena/data/view/ERP002217.

### Nontargeted Metabolite Profiling and Data Processing Methods

One hundred milligrams of lyophilized stool sample were extracted two times with 1 ml of 3∶2∶2 isopropanol:acetonitrile:water spun at 14,000 rpm for 5 minutes and the supernatants were combined. The extract was dried using a speedvac, resuspended in 50 µL of pyridine containing 15 mg/mL of methoxyamine hydrochloride, incubated at 60°C for 45 min, sonicated for 10 min, and incubated for an additional 45 min at 60°C. Next, 50 µL of N-methyl-N-trimethylsilyltrifluoroacetamide with 1% trimethylchlorosilane (MSTFA +1% TMCS, Thermo Scientific) was added and samples were incubated at 60°C for 30 min, centrifuged at 3000×g for 5 min, cooled to room temperature, and 80 µL of the supernatant was transferred to a 150 µL glass insert in a GC-MS autosampler vial. Metabolites were detected using a Trace GC Ultra coupled to a Thermo DSQ II (Thermo Scientific). Samples were injected in a 1∶10 split ratio twice in discrete randomized blocks. Separation occurred using a 30 m TG-5MS column (Thermo Scientific, 0.25 mm i.d., 0.25 µm film thickness) with a 1.2 mL/min helium gas flow rate, and the program consisted of 80°C for 30 sec, a ramp of 15°C per min to 330°C, and an 8 min hold. Masses between 50–650 *m/z* were scanned at 5 scans/sec after electron impact ionization. For each sample, a matrix of molecular features as defined by retention time and mass (*m/z*) was generated using XCMS software [25]. Features were normalized to total ion current, and the relative quantity of each molecular feature was determined by the mean area of the chromatographic peak among replicate injections (n = 2). Molecular features were formed into peak groups using AMDIS software [26], and spectra were screened in the National Institute for Technology Standards (www.nist.gov) and Golm (http://gmd.mpimp-golm.mpg.de/) metabolite databases for identifications.

*SCFA determination.* Stool samples were extracted for short chain fatty acids by mixing 1 g of frozen feces with acidified water (pH 2.5) and sonicated for 10 min. Samples were centrifuged and filtered through 0.45 µM nylon filters and stored at −80°C prior to analysis. The samples were analyzed using a Trace GC Ultra coupled to a Thermo DSQ II scanning from *m/z* 50–300 at a rate of 5 scans/second in electron impact mode. Samples were injected at a 10∶1 split ratio, and the inlet was held at 22°C and transfer line was held at 230°C. Separation was achieved on a 30 m TG-WAX-A column (Thermo Scientific, 0.25 mm ID, 0.25 µm film thickness) using a temperature program of 100°C for 1 min, ramped at 8°C per minute to 180°C, held at 180°C for one minute, ramped to 200°C at 20°C/minute, and held at 200°C for 5 minutes. Helium carrier flow was held at 1.2 mL per minute. Peak areas were integrated by Thermo Quan software using selected ions for each of the short chain fatty acids, and areas were normalized to total signal.

### Statistical Analysis

Differences in bacterial phylotypes and global metabolites between samples from healthy individuals and colon cancer patients were determined using AMOVA and student’s t-tests with a significance cutoff of <0.01. Phylotypes and metabolites that were significantly different between groups were further refined by removing markers that had fewer than 25 total reads (bacteria) or borderline background signals (metabolites) or that were present in fewer than 3 individual samples. Short chain fatty acid concentrations were determined in two separate chromatographic runs, so a weighted mean was calculated for each quantified compound and statistical differences between stool samples from healthy individuals and colon cancer patients were determined using a mixed model ANOVA with experiment representing a random effect and disease status as a fixed effect (XLSTAT 2011.1, Addinsoft Corp, Paris, France). Correlations between metabolites and bacteria were determined using Pearson’s r with a moderate correlation denoted by an r≥0.50 and a strong correlation denoted by an r≥0.70.

## Results and Discussion

### Alpha and Beta Diversity in Stool Biota

Typical community descriptors of alpha diversity for molecular microbial data include actual and estimated OTU richness, and indices of population diversity and evenness. In systems where pathogens are introduced (e.g. *Helicobacter pylori)*, there are marked decreases in estimates of diversity and evenness [27] suggesting that these indices may be useful predictors of infection. We examined these parameters in stool samples from healthy individuals and those with CRC to see if they could be used as predictors of disease state.We observed no significant differences at the 3% genetic distance in the average diversity or evenness of stool microbial communities from healthy individuals compared to those with CRC (Table S1). The average coverage obtained from 1250 reads per sample was 84% and 86% in healthy and colon cancer samples respectively. The average effective diversity of each group suggested a trend toward higher bacterial diversity in stool samples of healthy individuals (S_H_ = 63_,_ S_D_ = 20) compared to those from CRC patients (S_H_ = 46_,_ S_D_ = 15); however, the inter-individual variation was too great to achieve statistical significance. Based on these data, we suggest that alpha diversity descriptors of stool microbiota are not indicative of disease state in CRC; although a limitation of this study is that only stool samples and not tissue mucosa were analyzed. However, despite inherent differences in stool and mucosal microbial communities our findings are consistent with other published reports of total bacterial diversity and evenness estimates between CRC and healthy stool and tissue/mucosasamples [10].

**Figure 1:**
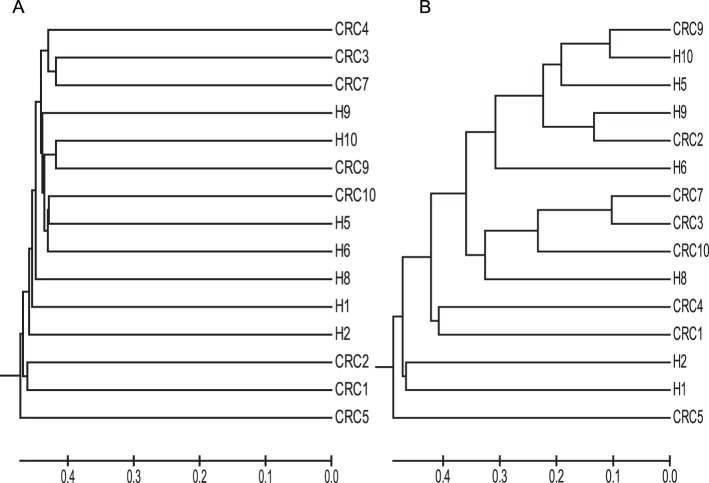
Using the 3% genetic distance, we observed no clustering of samples according to total stool microbial communities based on disease status of the sample donor using either the unweighted measure Jaccard similarity (A) or the weighted Theta_YC_ distance (B).

This inter-individual variation was also apparent in estimates of beta diversity, where a low degree of similarity in overall microbial community composition between individuals was observed as determined using the unweighted Jaccard distance (J_class_) to compare community membership (Figure 1A) and Yue and Clayton’s [28] index (Θ_YC_) to compare community structures (Figure 1B). Because of this variation, no patterns in the overall community composition were noted between stool samples from CRC patients and healthy individuals.

### Taxonomic Differences between CRC and Healthy Stool Samples

**Figure 2:**
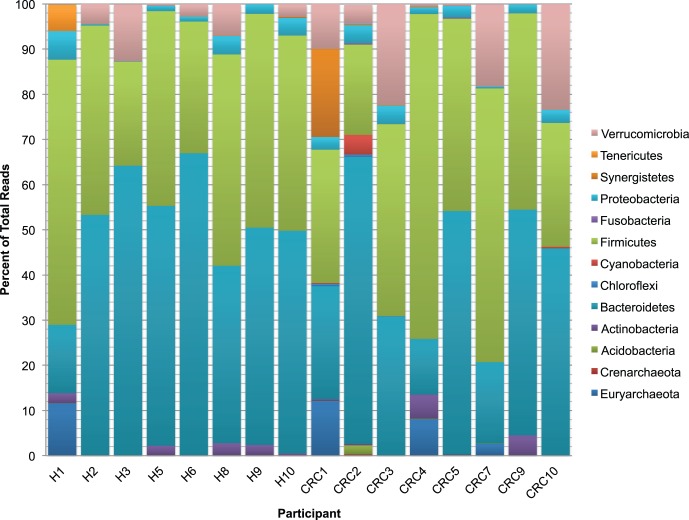
Phyla-level microbial classification of bacteria from individual stool samples. H sample numbers indicate samples from healthy adults while the C designation signifies samples from colon cancer patients.

The disease status of study participants did not drive overall community structure of the stool microbiota, and the composition and relative abundance of the major phyla were similar, although there was a non-significant trend towards higher Verrucomicrobia in samples from colon cancer patients (Figure 2). There were also higher levels of Synergetes in the cancer group, but this was driven by a single individual with an extremely high proportion of this phyla and was not representative of the entire sequenced cancer population. However, at the genus/species level there were a number of OTU’s that were significantly under-represented in the stool of colon cancer patients compared to healthy individuals (Table 2). These include several Gram-negative *Bacteroides* and *Prevotella spp*. that have previously been isolated from human stool, but are not well characterized with regards to their role in intestinal function or general health. Two of the *Prevotella* species identified were not only under-represented, but were completely absent from the colon cancer samples analyzed. *Prevotella* was a dominant genera reported in stool from children in a rural community in Burkina Faso but absent from a cohort of Italian children, and the study authors hypothesized that *Prevotella* helped maximize energy harvest from a plant-based diet [29]. Therefore, it is possible that the higher levels of *Prevotella* in the healthy cohort may reflect differences in the intake of fiber and other plant compounds compared to the individuals with colon cancer. At the genus level, Shen et al [30] found the *Bacteroides spp.* to be enriched in colonic tissue from healthy individuals when compared to adenoma tissue. Lachnospiracae and members of the genera *Dorea* and *Ruminococcus* were also previosly reported as dominant phylotypes driving differences between healthy and cancerous tissue samples [13]. The other OTUs that we identified such as the *Dialister spp.* and *Megamonas spp*. have not previously been reported in association with colon cancer; however, decreased populations of *Dialister invisus* have been reported in Crohn’s disease [31].

**Table 2:** Bacterial species that were significantly more abundant in the stool of healthy individuals compared to CRC patients.

Bacterial Species	Avg. Healthy (%)	Avg. CRC (%)	Fold Change	p value
*Bacteroides finegoldii*	0.74	0.29	2.5	0.0032
*Bacteroides intestinalis*	0.53	0.19	2.9	0.0063
*Prevotella copri*	4.09	0	40	0.0000
*Prevotella oris*	1.64	0	16	0.0001
*Ruminococcus obeum*	0.62	0.34	1.8	0.0009
*Dorea formicigenerans*	0.24	0.08	2.9	0.0001
*Lachnobacterium bovis*	1.20	0.62	1.9	0.0002
*Lachnospira pectinoschiza*	0.54	0.21	2.6	0.0005
*Pseudobutyrivibrio ruminis*	0.39	0.12	3.2	0.0000
*Bacteroides capillosus*	0.23	0.10	2.2	0.0057
*Ruminococcus albus*	0.36	0.03	10.3	0.0008
*Dialister invisus*	3.45	0.07	48.7	0.0000
*Dialister pneumosintes*	0.48	0.01	52.6	0.0000
*Megamonas hypermegale*	0.24	<0.01	44.5	0.0001

There were fewer identifiable bacteria that were over-represented in the colon cancer population (Table 3). Most notably, we observed that the mucin-degrading bacteria, *Akkermansia muciniphila*, which represented a relatively large percentage of the total sequences, was present in a significantly greater proportion in the feces of colon cancer patients. This bacterium is a common member of the colonic microbiota and was recently shown to be reduced in irritable bowel syndrome and Crohn’s Disease [32]; however a more recent report showed increased *A. muciniphila* in ulcerative colitis-associated pouchitis [33]. Two types of mucins, MUC1and MUC5AC, are reportedely overexpressed in colon cancers [34], suggesting that our observed CRC-related increases in *A. muciniphila* populations may be due to increased substrate availability. *Citrobacter farmeri*, which can utilize citrate as a sole carbon source was also higher in samples from colon cancer patients, but represented a much smaller proportion of the total bacterial sequences. *Citrobacter farmeri* is among a group of gut bacteria that includes multiple pathogenic species like *Salmonella* and *Shigella,* and which has arylamine *N*-acetyltransferase activity that may be involved in activation of carcinogens and xenobiotic metabolism [35].

**Table 3:** Bacterial species significantly over-represented in CRC stool samples.

Bacterial Species	Avg. Healthy (%)	Avg. CRC (%)	Fold Change	p value
*Acidaminobacter unclassified*	0.05	0.39	7.7	0.0045
*Phascolarctobacterium unclassified*	3.31	11.0	3.2	0.0000
*Citrobacter farmeri*	0.08	0.37	4.6	0.0050
*Akkermansia muciniphila*	3.54	12.8	3.6	0.0032

Age and BMI represent other factors that play a role in shaping the intestinal microbial communities. Several reports have demonstrated a correlation between the ratio of Bacteroidetes to Firmicutes and obesity [1]. We conducted linear regressions between the relative abundance of each of the taxa that significantly differed between CRC and healthy stools (see Tables 2 and 3) and BMI and saw no significant correlations (Table S2). In addition, aging has been associated with a decrease in protective commensal anaerobes, such as *Feacalibacterium prausnitzii,* and an increase in *E. coli* [*36*]. We did find a negative correlation between the age of participants and *Dorea formicagens* (R^2^ = 0.354; p = 0.041) and *Ruminococcus obeum* (R^2^ = 0.434; p = 0.020), both members of the Clostridium XIVa group, suggesting that differences between cohorts with regard to these two species may be a result of differences in the mean age of participants in each group rather than CRC disease status. To our knowledge, a decline in the population of Clostridium XIVa group members has not been previously associated with aging, but has been associated with dysbiosis related to intestinal inflammatory conditions such as Crohn’s disease [37]. None of the other bacterial taxa identified were correlated with age (Table S3). Therefore, we conclude that the majority of taxa that significantly differed in stool samples between healthy and CRC cohorts was a result of disease status and not of differences in age or BMI.

### Short Chain Fatty Acid Analysis

**Figure 3:**
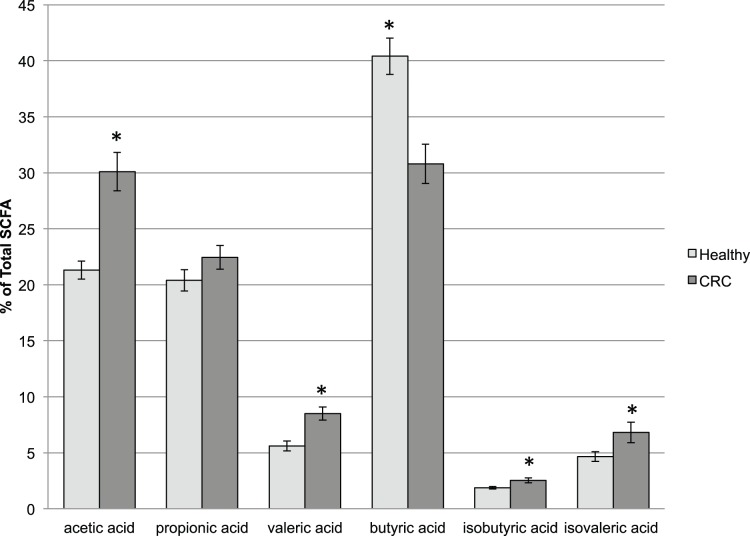
The relative proportion of bacterially-produced short chain fatty acids (SCFA) differed significantly between stool of healthy adults and individuals with CRC. Acetic acid, valeric acid, isobutyric acid, and isovaleric acid concentrations were proportionately higher while the anti-proliferative SCFA, butyric acid was significantly lower.

Short chain fatty acids (SCFA), particularly butyrate, are widely studied microbial metabolites reported to have anti-tumorigenic effects [38]. SCFA’s are readily absorbed and utilized in host tissues so detection in stool is typically considered an indication of production in excess of that which can be utilized by the host [29]. We and others [10], [13] have observed that species of butyrate producing bacteria, such as *Ruminococcus spp*. and *Pseudobutyrivibrio ruminis*, were lower in stool samples from CRC patients compared to healthy controls. Therefore, we quantified several short chain fatty acids from frozen stool samples. The three major SCFAs produced as microbial metabolites, acetate, propionate, and butyrate, were all detected as were valeric, isobutyric, isovaleric, caproic, and heptanoic acids. Among these, acetic and valeric acids were significantly higher in stool samples from CRC patients (p<0.0001 and p = 0.024 respectively) while butyric acid was significantly higher in the feces of healthy individuals (p<0.0001; Figure 3). No differences in propionic acid were detected between the two groups. Butyrate is regarded as one of the most important nutrients for normal colonocytes, and alone or in combination with propionate it has been shown to reduce proliferation and induce apotosis in human colon carcinomas [39]. Although acetate is an important SCFA for maintaining colonic health and as a precurser molecule for endogenous cholesterol production, elevated levels of this metabolite have previously been associated with CRC in humans [40]. Acetate can be used to produce butyrate and proportional differences in these metabolites between CRC and healthy samples may reflect a depletion of colonic microbes that can carry out this reaction in CRC samples or it may be a result of degradation of butyrate to acetate under low colonic pH associated with CRC. We also observed significantly higher relative concentrations of isobutyric (p<0.0001) and isovaleric acid (p = 0.002) in samples from individuals with CRC (Figure 3). These two SCFA’s result from bacterial metabolism of branched chain amino acids valine and leucine, which were also higher in CRC stool samples (Table 4), and may account for the significant increases observed in these two SCFAs in the CRC population.

**Table 4:** Candidate stool metabolites identified from GC-MS chromatograms that differ between CRC and healthy individuals.

Candidate	Chemical Class	% changein CRC	*p value*
Alanine	Amino Acid	74.0	<0.001
Glutamate	Amino Acid	76.1	<0.0001
Glycine	Amino Acid	72.3	<0.01
Aspartic acid	Amino Acid	82.2	<0.0001
Leucine	Amino Acid	61.0	<0.005
Lysine	Amino Acid	59.2	<0.05
Proline	Amino Acid	85.0	<0.001
Serine	Amino Acid	41.6	<0.005
Threonine	Amino Acid	79.7	<0.001
Valine	Amino Acid	73.0	<0.001
Phenylalanine	Amino Acid	77.3	<0.001
Benzeneacetic Acid	Carboxylic Acid	42.5	<0.005
Propionic acid	Short Chain Fatty Acid	74.2	<0.001
Myristic Acid	Saturated Fatty Acid	61.3	<0.001
Pantothenic acid	Vitamin B5	46.5	<0.01
Cholesterol derivative	Steroid	45.2	<0.005
Oleic acid*	unsaturated fatty acid	−74.6	<0.05
Linoleic acid*	unsaturated fatty acid	−67.3	<0.005
Elaidic acid*	unsaturated fatty acid	−45.5	<0.005
Glycerol	Polyol	−53.3	<0.005
Monooleoylglycerol	Polyol derivative	−55.4	<0.01
Ursodeoxycholic acid	Bile acid	−63.1%	<0.005

* Fatty acid identifications were conducted at a level that does not distinguish bond placement.

### Global Stool Metabolites

**Figure 4:**
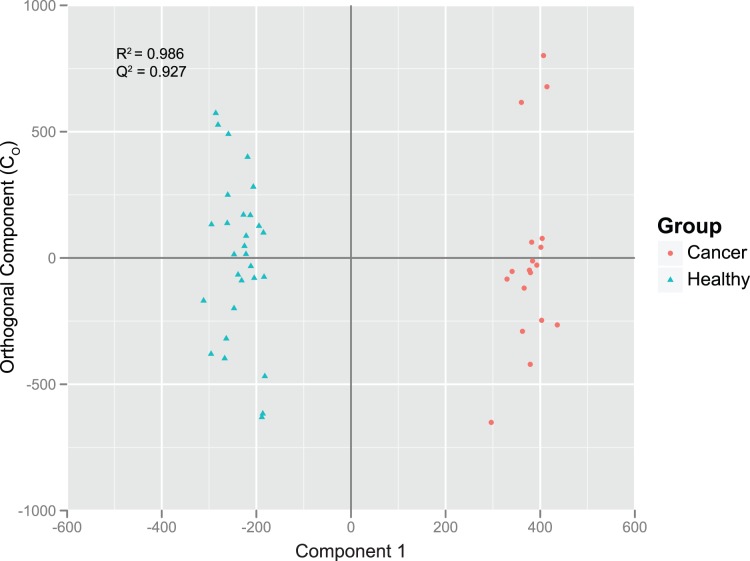
OPLS-DA scores plot generated from global GC-MS profiles differentiate stool metabolites from CRC patients and healthy adults.

Stool samples allow for evaluation of bacteria residing in the intestinal lumen, and therefore, stool small molecules are considered to result from co-metabolism or metabolic exchange between microbes and host cells [13]. Global metabolite profiling performed herein on lyophilized stool samples provided insights into the relationship between microbial populations and metabolites, and lend to the identification of novel CRC metabolic biomarkers. The supervised multivariate analysis technique, Orthogonal Projection to Latent Structures-Discriminant Analysis (OPLS-DA), which facilitates interpretation by separately modeling predictive and orthogonal (non-predictive) variables, was used to determine if non-targeted GC-MS profiles were predictive of disease state of the donor. The OPLS-DA demonstrated satisfactory modeling and predictive capabilities for this dataset (R2Y = 0.986; QY2 = 0.927), revealing a distinct separation between stool metabolic features of the two groups (Figure 4), suggesting that presence or absence of CRC is an important factor driving the variability in stool metabolites.

Compared to healthy controls, stool metabolome analysis revealed 11 amino acids that showed a 41–80% increase in stool samples of individuals with CRC (Table 4). Reasons that could account for this CRC-associated increase in amino acid concentrations may include, but not be limited to differences in protein consumption patterns, inflammation-induced reduction in nutrient absorption, and increased autophagy associated with tumor cells resulting in accumulation of free amino acid pools [41]. Microbial degradation of dietary proteins in the distal colon is a putreficative process that results in the production of toxic amines, and may account for the increased free amino acids we observed in CRC stool samples. An increased concentration of all amino acids except glutamine was previously reported in stomach and colon tumor tissues compared to healthy tissue [42]. The authors hypothesized that tumor cells may exhibit increased glutaminase activity resulting in glutamine conversion to glutamate. Consistent with these findings, we also saw a large increase, approximately 76%, in glutamate without a corresponding increase in glutamine in stool samples from colon cancer patients. Another recent study using NMR to identify and detect metabolites from stool water extracts from healthy and CRC samples showed that the CRC samples had approximately 1.5-fold higher levels of cysteine, proline, and leucine [43]. The increased concentrations of proline, serine, and threonine that were observed in CRC samples could also be the result from degradation of intestinal mucins, which are primarily comprised of glycoproteins rich in these amino acids [44]. This is consistent with the enrichment of *Akkermansia muciniphila*, a mucin-degrading bacteria, observed in CRC stool samples; although we saw no strong correlations between the relative proportion of these bacteria and specific amino acid concentrations.

There were higher levels of glycerol as well as several unsaturated fatty acids detected in the stool samples of healthy individuals. Human cancer cells have a known transport system for the uptake of glycerol, suggesting stool glycerol may be lower in CRC because it is being taken up by the tumor cells. Alternatively, bacterial lipases present in healthy individuals may facilitate the metabolism of dietary and endogenously produced triacylglycerols, resulting in the final degradation products of glycerol and free fatty acids. In addition to glycerol, fatty acids most closely matching metabolomic signatures for linoleic acid, and stereoisomers of oleic acid were also higher in controls (Table 4). Finally, ursodeoxycholic acid (UDCA), a secondary bile acid produced by intestinal bacteria was approximately 63% higher in healthy individuals compared to CRC. While several bile acids such as lithicolic acid and deoxycholic acid have been associated with tumorigenesis, UDCA has shown chemopreventive effects in preclinical and animal models of CRC [45].

**Figure 5:**
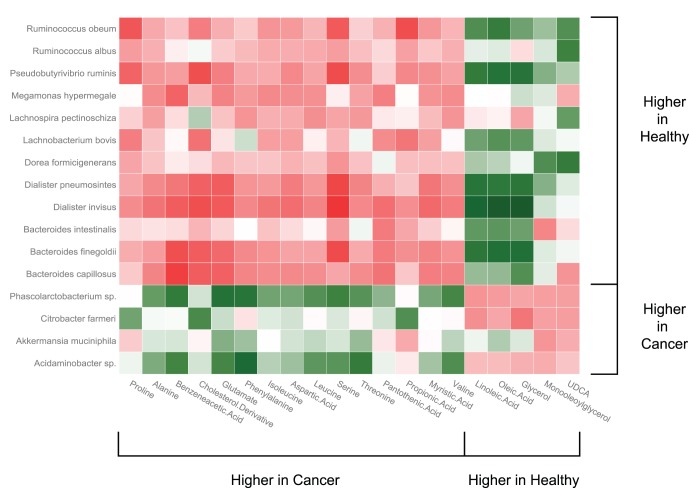
A heat map showing Pearson’s correlations between groups of metabolites and bacterial genera/species that significantly differed between CRC patients and healthy adults. Green boxes indicate positive associations and red boxes indicate negative associations.

Correlation analysis of the microbiome and metabolome data revealed strong associations between some members of the stool microbiota and candidate metabolites. *Bacteroides finegoldii,* two *Dialister spp.*, and *P. ruminis* were strongly correlated, and *Bacteroides intestinalis* and *Ruminococcus obeum* were moderately correlated with increased stool free fatty acids and glycerol (Figure 5). These same bacteria were inversely associated with a cholesterol derivative and one or more of the amino acids that were overrepresented in stool samples from CRC patients. The two *Ruminococcus spp.* also showed a strong positive correlation with the presence of UDCA, in concurrence with previous reports that *Ruminoccoccus* species exhibit 7α- and 7β-hydroxysteroid dehydrogenase activities to produce this metabolite [46]. Two of the bacterial genera overrepresented in CRC, *Phascolarctobacterium* and *Acidiminobacter* showed a strong positive association with the amino acids phenylalanine and glutamate, and were moderately correlated with increased serine and threonine (Figure 5). Glutamate can be utilized by these bacteria as a substrate, but their association with serine and threonine could also be indicative of involvement in mucin degradation or putrificative processes in the colon and warrant further study.

Extensive attempts to characterize CRC microbiota have led to new hypotheses as to how the gut microbiota influences CRC development. One hypothesis suggests that there are “driver bacteria” with pro-carcinogenic features that contribute to tumor development and “passenger bacteria” that may outcompete drivers to flourish in the tumor environment as the cancer progresses [47]. Available metabolites, those produced by bacteria and those that they utillize as substrates will largely drive these host-microbiome interactions. Integrating metabolome and microbiome datasets is a novel approach towards finding new directions to functionally characterize the microbiota in terms of their metabolic activity relative to cancer will greatly assist in our understanding of this complex host-microbe interaction.

## Supporting Information

Table S1 **Comparison of observed and estimated OTU richness and diversity and evenness indices between microbial communities from stool of CRC patients and healthy adults.**
(DOCX)

Table S2 **Linear regressions of selected bacterial taxa with participant BMI.**
(DOCX)

Table S3 **Linear regressions of selected bacterial taxa with participant age.**
(DOCX)

## References

[1] Turnbaugh PJ, Ley RE, Mahowald MA, Magrini V, Mardis ER, et al (2006) An obesity-associated gut microbiome with increased capacity for energy harvest. Nature 444: 1027–1131. 17183312 10.1038/nature05414

[2] Neish AS (2009) Microbes in Gastrointestinal Health and Disease. Gastroenterology 136: 65–80. 19026645 10.1053/j.gastro.2008.10.080 PMC2892787

[3] Kelly D, Conway S, Aminov R (2005) Commensal gut bacteria: mechanisms of immune modulation. Trends in Immunology 26: 326–333. 15922949 10.1016/j.it.2005.04.008

[4] Selgrad M, Malfertheiner P, Fini L, Goel A, Boland CR, et al (2008) The role of viral and bacterial pathogens in gastrointestinal cancer. Journal of Cellular Physiology 216: 378–388. 18338378 10.1002/jcp.21427 PMC2855192

[5] Klein RS, Catalano MT, Edberg SC, Casey JI, Steigbigel NH, et al (1979) Streptococcus bovis septicemia and carcinoma of the colon. Ann Intern Med 91: 560–562. 484953 10.7326/0003-4819-91-4-560

[6] Leport C, Bure J, Leport J, Vilde JL (1987) Incidence of colonic lesions in Streptococcus bovis and enterococcal endocarditis. Lancet 1: 748–749. 10.1016/s0140-6736(87)90391-6 2882164

[7] Hill MJ, Drasar BS, Aries V, Crowther JS, Hawksworth G, et al (1971) BACTERIA AND ÆTIOLOGY OF CANCER OF LARGE BOWEL. The Lancet 297: 95–100. 10.1016/s0140-6736(71)90837-3 4099643

[8] Maclennan R, Jensen OM (1977) Dietary fibre, transit-time, faecal bacteria, steroids, and colon cancer in two Scandinavian populations. Report from the International Agency for Research on Cancer Intestinal Microecology Group. Lancet 2: 207–211. 69826

[9] Moore WE, Moore LH (1995) Intestinal floras of populations that have a high risk of colon cancer. Applied and Environmental Microbiology 61: 3202–3207. 7574628 10.1128/aem.61.9.3202-3207.1995 PMC167598

[10] Sobhani I, Tap J, Roudot-Thoraval F, Roperch JP, Letulle S, et al (2011) Microbial Dysbiosis in Colorectal Cancer (CRC) Patients. PLoS ONE 6: e16393. 21297998 10.1371/journal.pone.0016393 PMC3029306

[11] Castellarin M, Warren RL, Freeman JD, Dreolini L, Krzywinski M, et al (2011) Fusobacterium nucleatum infection is prevalent in human colorectal carcinoma. Genome Research 22: 299–306. 22009989 10.1101/gr.126516.111 PMC3266037

[12] Kostic AD, Gevers D, Pedamallu CS, Michaud M, Duke F, et al (2011) Genomic analysis identifies association of Fusobacterium with colorectal carcinoma. Genome Research 22: 292–298. 22009990 10.1101/gr.126573.111 PMC3266036

[13] Chen W, Liu F, Ling Z, Tong X, Xiang C (2012) Human Intestinal Lumen and Mucosa-Associated Microbiota in Patients with Colorectal Cancer. PLoS ONE 7: e39743. 22761885 10.1371/journal.pone.0039743 PMC3386193

[14] Marchesi JR, Dutilh BE, Hall N, Peters WHM, Roelofs R, et al (2011) Towards the Human Colorectal Cancer Microbiome. PLoS ONE 6: e20447. 21647227 10.1371/journal.pone.0020447 PMC3101260

[15] Huycke MM, Gaskins HR (2004) Commensal Bacteria, Redox Stress, and Colorectal Cancer: Mechanisms and Models. Experimental Biology and Medicine 229: 586–597. 15229352 10.1177/153537020422900702

[16] Nicholson JK, Lindon JC (2008) Systems biology: Metabonomics. Nature 455: 1054–1056. 18948945 10.1038/4551054a

[17] Nicholls AW, Mortishire-Smith RJ, Nicholson JK (2003) NMR Spectroscopic-Based Metabonomic Studies of Urinary Metabolite Variation in Acclimatizing Germ-Free Rats. Chemical Research in Toxicology 16: 1395–1404. 14615964 10.1021/tx0340293

[18] Martin F-PJ, Sprenger N, Montoliu I, Rezzi S, Kochhar S, et al (2010) Dietary Modulation of Gut Functional Ecology Studied by Fecal Metabonomics. Journal of Proteome Research 9: 5284–5295. 20806900 10.1021/pr100554m

[19] Kinross J, Darzi A, Nicholson J (2011) Gut microbiome-host interactions in health and disease. Genome Medicine 3: 14. 21392406 10.1186/gm228 PMC3092099

[20] Jacobs DM, Deltimple N, van Velzen E, van Dorsten FA, Bingham M, et al (2008) 1H NMR metabolite profiling of feces as a tool to assess the impact of nutrition on the human microbiome. NMR in Biomedicine 21: 615–626. 18085514 10.1002/nbm.1233

[21] Fierer N, Hamady M, Lauber CL, Knight R (2008) The influence of sex, handedness, and washing on the diversity of hand surface bacteria. Proceedings of the National Academy of Sciences 105: 17994–17999. 10.1073/pnas.0807920105 PMC2584711 19004758

[22] Schloss PD, Westcott SL, Ryabin T, Hall JR, Hartmann M, et al (2009) Introducing mothur: Open-Source, Platform-Independent, Community-Supported Software for Describing and Comparing Microbial Communities. Applied and Environmental Microbiology 75: 7537–7541. 19801464 10.1128/AEM.01541-09 PMC2786419

[23] Wang Q, Garrity GM, Tiedje JM, Cole JR (2007) Naïve Bayesian Classifier for Rapid Assignment of rRNA Sequences into the New Bacterial Taxonomy. Applied and Environmental Microbiology 73: 5261–5267. 17586664 10.1128/AEM.00062-07 PMC1950982

[24] Huse SM, Welch DM, Morrison HG, Sogin ML (2010) Ironing out the wrinkles in the rare biosphere through improved OTU clustering. Environmental Microbiology 12: 1889–1898. 20236171 10.1111/j.1462-2920.2010.02193.x PMC2909393

[25] Smith CA, Want EJ, O'Maille G, Abagyan R, Siuzdak G (2006) XCMS: Processing Mass Spectrometry Data for Metabolite Profiling Using Nonlinear Peak Alignment, Matching, and Identification. Analytical Chemistry 78: 779–787. 16448051 10.1021/ac051437y

[26] Stein SE (1999) An integrated method for spectrum extraction and compound identification from gas chromatography/mass spectrometry data. Journal of the American Society for Mass Spectrometry 10: 770–781.

[27] Andersson AF, Lindberg M, Jakobsson H, Bäckhed F, Nyrén P, et al (2008) Comparative Analysis of Human Gut Microbiota by Barcoded Pyrosequencing. PLoS ONE 3: e2836. 18665274 10.1371/journal.pone.0002836 PMC2475661

[28] Yue JC, Clayton MK (2005) A Similarity Measure Based on Species Proportions. Communications in Statistics - Theory and Methods 34: 2123–2131.

[29] De Filippo C, Cavalieri D, Di Paola M, Ramazzotti M, Poullet JB, et al (2010) Impact of diet in shaping gut microbiota revealed by a comparative study in children from Europe and rural Africa. Proceedings of the National Academy of Sciences 107: 14691–14696. 10.1073/pnas.1005963107 PMC2930426 20679230

[30] Shen XJ, Rawls JF, Randall TA, Burcall L, Mpande C, et al (2010) Molecular characterization of mucosal adherent bacteria and associations with colorectal adenomas. Gut Microbes 1: 138–147. 20740058 10.4161/gmic.1.3.12360 PMC2927011

[31] Joossens M, Huys G, Cnockaert M, De Preter V, Verbeke K, et al.. (2011) Dysbiosis of the faecal microbiota in patients with Crohn's disease and their unaffected relatives. Gut. 10.1136/gut.2010.223263 21209126

[32] Png CW, Linden SK, Gilshenan KS, Zoetendal EG, McSweeney CS, et al (2010) Mucolytic Bacteria With Increased Prevalence in IBD Mucosa Augment In Vitro Utilization of Mucin by Other Bacteria. Am J Gastroenterol 105: 2420–2428. 20648002 10.1038/ajg.2010.281

[33] Zella GC, Hait EJ, Glavan T, Gevers D, Ward DV, et al (2011) Distinct microbiome in pouchitis compared to healthy pouches in ulcerative colitis and familial adenomatous polyposis. Inflammatory Bowel Diseases 17: 1092–1100. 20845425 10.1002/ibd.21460

[34] Byrd JC, Bresalier RS (2004) Mucins and mucin binding proteins in colorectal cancer: Colorectal Cancer. Cancer and Metastasis Reviews 23: 77–99. 15000151 10.1023/a:1025815113599

[35] Deloménie C, Fouix S, Longuemaux S, Brahimi Nm, Bizet C, et al (2001) Identification and Functional Characterization of Arylamine N-Acetyltransferases in Eubacteria: Evidence for Highly Selective Acetylation of 5-Aminosalicylic Acid. Journal of Bacteriology 183: 3417–3427. 11344150 10.1128/JB.183.11.3417-3427.2001 PMC99640

[36] Bartosch S, Fite A, Macfarlane GT, McMurdo MET (2004) Characterization of Bacterial Communities in Feces from Healthy Elderly Volunteers and Hospitalized Elderly Patients by Using Real-Time PCR and Effects of Antibiotic Treatment on the Fecal Microbiota. Applied and Environmental Microbiology 70: 3575–3581. 15184159 10.1128/AEM.70.6.3575-3581.2004 PMC427772

[37] Sartor RB (2008) Therapeutic correction of bacterial dysbiosis discovered by molecular techniques. Proceedings of the National Academy of Sciences 105: 16413–16414. 10.1073/pnas.0809363105 PMC2575433 18948599

[38] Williams EA, Coxhead JM, Mathers JC (2003) Anti-cancer effects of butyrate: use of micro-array technology to investigate mechanisms. Proceedings of the Nutrition Society 62: 107–115. 12740065 10.1079/PNS2002230

[39] Matthews GM, Howarth GS, Butler RN (2012) Short-Chain Fatty Acids Induce Apoptosis in Colon Cancer Cells Associated with Changes to Intracellular Redox State and Glucose Metabolism. Chemotherapy 58: 102–109. 22488147 10.1159/000335672

[40] Weaver GA, Krause JA, Miller TL, Wolin MJ (1988) Short chain fatty acid distributions of enema samples from a sigmoidoscopy population: an association of high acetate and low butyrate ratios with adenomatous polyps and colon cancer. Gut 29: 1539–1543. 3209110 10.1136/gut.29.11.1539 PMC1433834

[41] Sato K, Tsuchihara K, Fujii S, Sugiyama M, Goya T, et al (2007) Autophagy Is Activated in Colorectal Cancer Cells and Contributes to the Tolerance to Nutrient Deprivation. Cancer Research 67: 9677–9684. 17942897 10.1158/0008-5472.CAN-07-1462

[42] Hirayama A, Kami K, Sugimoto M, Sugawara M, Toki N, et al (2009) Quantitative Metabolome Profiling of Colon and Stomach Cancer Microenvironment by Capillary Electrophoresis Time-of-Flight Mass Spectrometry. Cancer Research 69: 4918–4925. 19458066 10.1158/0008-5472.CAN-08-4806

[43] Monleón D, Morales JM, Barrasa A, López JA, Vázquez C, et al (2009) Metabolite profiling of fecal water extracts from human colorectal cancer. NMR in Biomedicine 22: 342–348. 19006102 10.1002/nbm.1345

[44] Byrd J, Bresalier R (2004) Mucins and mucin binding proteins in colorectal cancer. Cancer and Metastasis Reviews 23: 77–99. 15000151 10.1023/a:1025815113599

[45] Akare S, Jean-Louis S, Chen W, Wood DJ, Powell AA, et al (2006) Ursodeoxycholic acid modulates histone acetylation and induces differentiation and senescence. International Journal of Cancer 119: 2958–2969. 17019713 10.1002/ijc.22231

[46] Lepercq P, Gérard P, Béguet F, Grill J-P, Relano P, et al (2011) Isolates from Normal Human Intestinal Flora but not Lactic Acid Bacteria Exhibit 7a- and b- Hydroxysteroid Dehydrogenase Activities. Microbial Ecology in Health and Disease 16: 1651–2235.

[47] Tjalsma H, Boleij A, Marchesi JR, Dutilh BE (2012) A bacterial driver–passenger model for colorectal cancer: beyond the usual suspects. Nat Rev Micro 10: 575–582. 10.1038/nrmicro2819 22728587

