# Supplementary material for: Stool Microbiome and Metabolome Differences between Colorectal Cancer Patients and Healthy Adults
Source: PLoS One. 2013 Aug 6;8(8):e70803. doi: 10.1371/journal.pone.0070803 (PMC3735522; doi:10.1371/journal.pone.0070803)
Supplement: Table S1 — Comparison of observed and estimated OTU richness and diversity and evenness indices between microbial communities from stool of CRC patients and healthy adults. (DOCX) [file pone.0070803.s001.docx]

**Table S1.** Comparison of observed and estimated OTU richness and diversity and evenness indices between microbial communities from stool of CRC patients and healthy adults.

|  |  |  |  |  | Shannon | | |  | Simpson | | |
| --- | --- | --- | --- | --- | --- | --- | --- | --- | --- | --- | --- |
| Group | nseqs | coverage | S_obs_ | S_chao_ | H’ | E_H_ | S_H_ |  | 1-D | E_D_ | S_D_ |
| Healthy | 1250 | 0.839 | 309 | 838 | 4.484 | 0.782 | 89 |  | 0.970 | 0.109 | 34 |
| Healthy | 1250 | 0.854 | 250 | 805 | 3.669 | 0.664 | 39 |  | 0.915 | 0.047 | 12 |
| Healthy | 1250 | 0.892 | 206 | 541 | 3.316 | 0.622 | 28 |  | 0.871 | 0.038 | 8 |
| Healthy | 1250 | 0.839 | 290 | 864 | 3.961 | 0.699 | 53 |  | 0.913 | 0.040 | 11 |
| Healthy | 1250 | 0.858 | 257 | 729 | 3.660 | 0.660 | 39 |  | 0.905 | 0.041 | 10 |
| Healthy | 1250 | 0.793 | 364 | 1243 | 4.663 | 0.791 | 106 |  | 0.973 | 0.103 | 38 |
| Healthy | 1250 | 0.817 | 325 | 1012 | 4.456 | 0.770 | 86 |  | 0.963 | 0.084 | 27 |
| Cancer | 1250 | 0.842 | 287 | 960 | 4.209 | 0.744 | 67 |  | 0.955 | 0.077 | 22 |
| Cancer | 1250 | 0.906 | 184 | 460 | 3.353 | 0.643 | 29 |  | 0.900 | 0.054 | 10 |
| Cancer | 1250 | 0.834 | 285 | 867 | 4.015 | 0.710 | 55 |  | 0.943 | 0.061 | 18 |
| Cancer | 1250 | 0.857 | 255 | 786 | 3.787 | 0.683 | 44 |  | 0.926 | 0.053 | 14 |
| Cancer | 1250 | 0.842 | 290 | 919 | 4.303 | 0.759 | 74 |  | 0.961 | 0.089 | 26 |
| Cancer | 1250 | 0.864 | 229 | 1127 | 3.582 | 0.659 | 36 |  | 0.925 | 0.058 | 13 |
| Cancer | 1250 | 0.861 | 263 | 670 | 3.822 | 0.686 | 46 |  | 0.919 | 0.047 | 12 |
| Cancer | 1250 | 0.867 | 232 | 803 | 3.674 | 0.675 | 39 |  | 0.926 | 0.058 | 14 |
| TTEST(H:C) |  | 0.24 | 0.18 | 0.73 | 0.41 | 0.54 | 0.27 |  | 0.90 | 0.78 | 0.44 |
