# Supplementary material for: Stool Microbiome and Metabolome Differences between Colorectal Cancer Patients and Healthy Adults
Source: PLoS One. 2013 Aug 6;8(8):e70803. doi: 10.1371/journal.pone.0070803 (PMC3735522; doi:10.1371/journal.pone.0070803)
Supplement: Table S2 — Linear regressions of selected bacterial taxa with participant BMI. (DOCX) [file pone.0070803.s002.docx]

**Table S2**. Linear regressions of selected bacterial taxa with participant BMI.

| **Bacterial taxa** | **R^2^ value** | **P value** |
| --- | --- | --- |
| *Bacteroides finegoldii* | 0.129 | 0.251 |
| *Bacteroides intestinalis* | 0.105 | 0.303 |
| *Ruminococcus obeum* | 0.038 | 0.543 |
| *Dorea formicigenerans* | 0.003 | 0.868 |
| *Lachnobacterium bovis* | 0.071 | 0.403 |
| *Lachnospira pectinoschiza* | 0.113 | 0.285 |
| *Pseudobutyrivibrio ruminis* | 0.013 | 0.724 |
| *Bacteroides capillosus* | 0.165 | 0.190 |
| *Ruminococcus albus* | 0.161 | 0.196 |
| *Dialister invisus* | 0.065 | 0.424 |
| *Dialister pneumosintes* | 0.001 | 0.905 |
| *Megamonas hypermegale* | 0.197 | 0.149 |
| *Acidaminobacter unclassified* | 0.016 | 0.699 |
| *Phascolarctobacterium unclassified* | 0.056 | 0.457 |
| *Citrobacter farmeri* | 0.218 | 0.126 |
| *Akkermansia muciniphila* | 0.064 | 0.428 |
