# Supplementary material for: Stool Microbiome and Metabolome Differences between Colorectal Cancer Patients and Healthy Adults
Source: PLoS One. 2013 Aug 6;8(8):e70803. doi: 10.1371/journal.pone.0070803 (PMC3735522; doi:10.1371/journal.pone.0070803)
Supplement: Table S3 — Linear regressions of selected bacterial taxa with participant age. (DOCX) [file pone.0070803.s003.docx]

**Table S3.** Linear regressions of selected bacterial taxa with participant age.

| **Bacterial taxa** | **R^2^ value** | **P value** |
| --- | --- | --- |
| *Bacteroides finegoldii* | 0.181 | 0.167 |
| *Bacteroides intestinalis* | 0.001 | 0.935 |
| *Ruminococcus obeum* | 0.434 | 0.02 |
| *Dorea formicigenerans* | 0.354 | 0.041 |
| *Lachnobacterium bovis* | 0.07 | 0.407 |
| *Lachnospira pectinoschiza* | 0.202 | 0.143 |
| *Pseudobutyrivibrio ruminis* | 0.209 | 0.135 |
| *Bacteroides capillosus* | 0.065 | 0.423 |
| *Ruminococcus albus* | 0.307 | 0.061 |
| *Dialister invisus* | 0.113 | 0.286 |
| *Dialister pneumosintes* | 0.136 | 0.238 |
| *Megamonas hypermegale* | 0.052 | 0.457 |
| *Acidaminobacter unclassified* | 0.115 | 0.218 |
| *Phascolarctobacterium unclassified* | 0.181 | 0.168 |
| *Citrobacter farmeri* | 0.188 | 0.16 |
| *Akkermansia muciniphila* | 0.018 | 0.678 |
